# Zika Virus Outside Africa

**DOI:** 10.3201/eid1509.090442

**Published:** 2009-09

**Authors:** Edward B. Hayes

**Affiliations:** Barcelona Centre for International Health Research, Barcelona, Spain

**Keywords:** Zika virus, Africa, flavivirus, transmission, virology, viruses, mosquito, synopsis

## Abstract

Zika virus (ZIKV) is a flavivirus related to yellow fever, dengue, West Nile, and Japanese encephalitis viruses. In 2007 ZIKV caused an outbreak of relatively mild disease characterized by rash, arthralgia, and conjunctivitis on Yap Island in the southwestern Pacific Ocean. This was the first time that ZIKV was detected outside of Africa and Asia. The history, transmission dynamics, virology, and clinical manifestations of ZIKV disease are discussed, along with the possibility for diagnostic confusion between ZIKV illness and dengue.The emergence of ZIKV outside of its previously known geographic range should prompt awareness of the potential for ZIKV to spread to other Pacific islands and the Americas.

## Abstract 2

This should be considered an emerging pathogen.

In April 2007, an outbreak of illness characterized by rash, arthralgia, and conjunctivitis was reported on Yap Island in the Federated States of Micronesia. Serum samples from patients in the acute phase of illness contained RNA of Zika virus (ZIKV), a flavivirus in the same family as yellow fever, dengue, West Nile, and Japanese encephalitis viruses. These findings show that ZIKV has spread outside its usual geographic range (*1*,*2*).

Sixty years earlier, on April 18, 1947, fever developed in a rhesus monkey that had been placed in a cage on a tree platform in the Zika Forest of Uganda (*3*). The monkey, Rhesus 766, was a sentinel animal in the Rockefeller Foundation’s program for research on jungle yellow fever. Two days later, Rhesus 766, still febrile, was brought to the Foundation’s laboratory at Entebbe and its serum was inoculated into mice. After 10 days all mice that were inoculated intracerebrally were sick, and a filterable transmissible agent, later named Zika virus, was isolated from the mouse brains. In early 1948, ZIKV was also isolated from *Aedes africanus* mosquitoes trapped in the same forest (*4*). Serologic studies indicated that humans could also be infected (*5*). Transmission of ZIKV by artificially fed *Ae. aegypti* mosquitoes to mice and a monkey in a laboratory was reported in 1956 (*6*).

ZIKV was isolated from humans in Nigeria during studies conducted in 1968 and during 1971–1975; in 1 study, 40% of the persons tested had neutralizing antibody to ZIKV (*7**–**9*). Human isolates were obtained from febrile children 10 months, 2 years (2 cases), and 3 years of age, all without other clinical details described, and from a 10 year-old boy with fever, headache, and body pains (*7**,**8*). From 1951 through 1981, serologic evidence of human ZIKV infection was reported from other African countries such as Uganda, Tanzania, Egypt, Central African Republic, Sierra Leone (*10*), and Gabon, and in parts of Asia including India, Malaysia, the Philippines, Thailand, Vietnam, and Indonesia (*10**–**14*). In additional investigations, the virus was isolated from *Ae. aegypti* mosquitoes in Malaysia, a human in Senegal, and mosquitoes in Côte d’Ivoire (*15**–**17*). In 1981 Olson et al. reported 7 people with serologic evidence of ZIKV illness in Indonesia (*11*). A subsequent serologic study indicated that 9/71 (13%) human volunteers in Lombok, Indonesia, had neutralizing antibody to ZIKV (*18*). The outbreak on Yap Island in 2007 shows that ZIKV illness has been detected outside of Africa and Asia (Figure 1).

**Figure 1:**
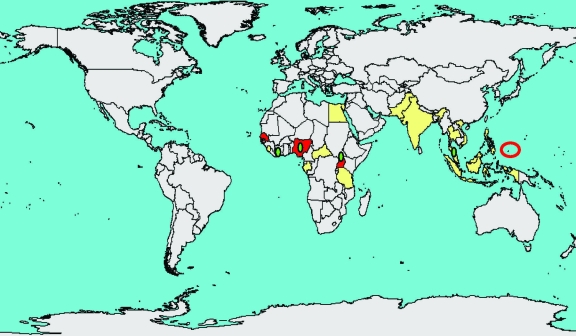
Approximate known distribution of Zika virus, 1947–2007. Red circle represents Yap Island. Yellow indicates human serologic evidence; red indicates virus isolated from humans; green represents mosquito isolates.

## Dynamics of Transmission

ZIKV has been isolated from *Ae. africanus*, *Ae. apicoargenteus*, *Ae. luteocephalus*, *Ae. aegypti*, *Ae vitattus*, and *Ae. furcifer* mosquitoes (*9**,**15**,**17**,**19*). *Ae. hensilii* was the predominant mosquito species present on Yap during the ZIKV disease outbreak in 2007, but investigators were unable to detect ZIKV in any mosquitoes on the island during the outbreak (*2*). Dick noted that *Ae. africanus* mosquitoes, which were abundant and infected with ZIKV in the Zika Forest, were not likely to enter monkey cages such as the one containing Rhesus 766 (*5*) raising the doubt that the monkey might have acquired ZIKV from some other mosquito species or through some other mechanism. During the studies of yellow fever in the Zika Forest, investigators had to begin tethering monkeys in trees because caged monkeys did not acquire yellow fever virus when the virus was present in mosquitoes (*5*). Thus, despite finding ZIKV in *Ae. Africanus* mosquitoes, Dick was not sure whether or not these mosquitoes were actually the vector for enzootic ZIKV transmission to monkeys.

Boorman and Porterfield subsequently demonstrated transmission of ZIKV to mice and monkeys by *Ae. aegypti* in a laboratory (*6*). Virus content in the mosquitoes was high on the day of artificial feeding, dropped to undetectable levels through day 10 after feeding, had increased by day 15, and remained high from days 20 through 60 (*6*). Their study suggests that the extrinsic incubation period for ZIKV in mosquitoes is ≈10 days. The authors cautioned that their results did not conclusively demonstrate that *Ae. aegypti* mosquitoes could transmit ZIKV at lower levels of viremia than what might occur among host animals in natural settings. Nevertheless, their results, along with the viral isolations from wild mosquitoes and monkeys and the phylogenetic proximity of ZIKV to other mosquito-borne flaviviruses, make it reasonable to conclude that ZIKV is transmitted through mosquito bites.

There is to date no solid evidence of nonprimate reservoirs of ZIKV, but 1 study did find antibody to ZIKV in rodents (*20*). Further laboratory, field, and epidemiologic studies would be useful to better define vector competence for ZIKV, to determine if there are any other arthropod vectors or reservoir hosts, and to evaluate the possibility of congenital infection or transmission through blood transfusion.

## Virology and Pathogenesis

**Figure 2:**
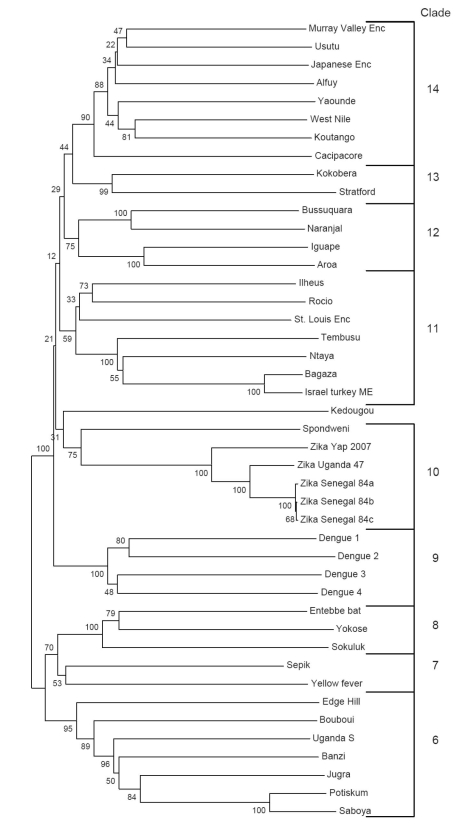
Phylogenetic relationship of Zika virus to other flaviviruses based on nucleic acid sequence of nonstructural viral protein 5, with permission from Dr Robert Lanciotti (*1*). Enc, encephalitis; ME, meningoencephalitis.

ZIKV is an RNA virus containing 10,794 nucleotides encoding 3,419 amino acids. It is closely related to Spondweni virus; the 2 viruses are the only members of their clade within the mosquito-borne cluster of flaviviruses (Figure 2) (*1**,**21**,**22*). The next nearest relatives include Ilheus, Rocio, and St. Louis encephalitis viruses; yellow fever virus is the prototype of the family, which also includes dengue, Japanese encephalitis, and West Nile viruses (*1**,**21*). Studies in the Zika Forest suggested that ZIKV infection blunted the viremia caused by yellow fever virus in monkeys but did not block transmission of yellow fever virus (*19**,**23*).

Information regarding pathogenesis of ZIKV is scarce but mosquito-borne flaviviruses are thought to replicate initially in dendritic cells near the site of inoculation then spread to lymph nodes and the bloodstream (*24*). Although flaviviral replication is thought to occur in cellular cytoplasm, 1 study suggested that ZIKV antigens could be found in infected cell nuclei (*25*). To date, infectious ZIKV has been detected in human blood as early as the day of illness onset; viral nucleic acid has been detected as late as 11 days after onset (*1**,**26*). The virus was isolated from the serum of a monkey 9 days after experimental inoculation (*5*). ZIKV is killed by potassium permanganate, ether, and temperatures >60°C, but it is not effectively neutralized with 10% ethanol (*5*).

## Clinical Manifestations

The first well-documented report of human ZIKV disease was in 1964 when Simpson described his own occupationally acquired ZIKV illness at age 28 (*27*). It began with mild headache. The next day, a maculopapular rash covered his face, neck, trunk, and upper arms, and spread to his palms and soles. Transient fever, malaise, and back pain developed. By the evening of the second day of illness he was afebrile, the rash was fading, and he felt better. By day three, he felt well and had only the rash, which disappeared over the next 2 days. ZIKV was isolated from serum collected while he was febrile.

In 1973, Filipe et al. reported laboratory-acquired ZIKV illness in a man with acute onset of fever, headache, and joint pain but no rash (*26*). ZIKV was isolated from serum collected on the first day of symptoms; the man’s illness resolved in ≈1 week.

Of the 7 ZIKV case-patients in Indonesia described by Olson et al. all had fever, but they were detected by hospital-based surveillance for febrile illness (*11*). Other manifestations included anorexia, diarrhea, constipation, abdominal pain, and dizziness. One patient had conjunctivitis but none had rash. The outbreak on Yap Island was characterized by rash, conjunctivitis, and arthralgia (*1**,**2*). Other less frequent manifestations included myalgia, headache, retroorbital pain, edema, and vomiting (*2*).

## Diagnosis

Diagnostic tests for ZIKV infection include PCR tests on acute-phase serum samples, which detect viral RNA, and other tests to detect specific antibody against ZIKV in serum. An ELISA has been developed at the Arboviral Diagnostic and Reference Laboratory of the Centers for Disease Control and Prevention (Ft. Collins, CO, USA) to detect immunoglobulin (Ig) M to ZIKV (*1*). In the samples from Yap Island, cross-reactive results in sera from convalescent-phase patients occurred more frequently among patients with evidence of previous flavivirus infections than among those with apparent primary ZIKV infections (*1**,**2*). Cross-reactivity was more frequently noted with dengue virus than with yellow fever, Japanese encephalitis, Murray Valley encephalitis, or West Nile viruses, but there were too few samples tested to derive robust estimates of the sensitivity and specificity of the ELISA. IgM was detectable as early as 3 days after onset of illness in some persons; 1 person with evidence of previous flavivirus infection had not developed IgM at day 5 but did have it by day 8 (*1*). Neutralizing antibody developed as early as 5 days after illness onset. The plaque reduction neutralization assay generally has improved specificity over immunoassays, but may still yield cross-reactive results in secondary flavivirus infections. PCR tests can be conducted on samples obtained less than 10 days after illness onset; 1 patient from Yap Island still had detectable viral RNA on day 11 (*1*). In general, diagnostic testing for flavivirus infections should include an acute-phase serum sample collected as early as possible after onset of illness and a second sample collected 2 to 3 weeks after the first.

## Public Health Implications

Because the virus has spread outside Africa and Asia, ZIKV should be considered an emerging pathogen. Fortunately, ZIKV illness to date has been mild and self-limited, but before West Nile virus caused large outbreaks of neuroinvasive disease in Romania and in North America, it was also considered to be a relatively innocuous pathogen (*28*). The discovery of ZIKV on the physically isolated community of Yap Island is testimony to the potential for travel or commerce to spread the virus across large distances. A medical volunteer who was on Yap Island during the ZIKV disease outbreak became ill and was likely viremic with ZIKV after her return to the United States (*2*). The competence of mosquitoes in the Americas for ZIKV is not known and this question should be addressed. Spread of ZIKV across the Pacific could be difficult to detect because of the cross-reactivity of diagnostic flavivirus antibody assays. ZIKV disease could easily be confused with dengue and might contribute to illness during dengue outbreaks. Recognition of the spread of ZIKV and of the impact of ZIKV on human health will require collaboration between clinicians, public health officials, and high-quality reference laboratories.

Given that the epidemiology of ZIKV transmission on Yap Island appeared to be similar to that of dengue, strategies for prevention and control of ZIKV disease should include promoting the use of insect repellent and interventions to reduce the abundance of potential mosquito vectors. Officials responsible for public health surveillance in the Pacific region and the United States should be alert to the potential spread of ZIKV and keep in mind the possible diagnostic confusion between ZIKV illness and dengue.
